# Zipf’s law revisited: Spoken dialog, linguistic units, parameters, and the principle of least effort

**DOI:** 10.3758/s13423-022-02142-9

**Published:** 2022-07-15

**Authors:** Guido M. Linders, Max M. Louwerse

**Affiliations:** Department of Cognitive Science & Artificial Intelligence, Tilburg University, Warandelaan 2, 5037 AB Tilburg, the Netherlands

**Keywords:** Zipf’s law, Principle of least effort, Dialog, Quantitative linguistics, Cognition

## Abstract

The ubiquitous inverse relationship between word frequency and word rank is commonly known as Zipf’s law. The theoretical underpinning of this law states that the inverse relationship yields decreased effort in both the speaker and hearer, the so-called principle of least effort. Most research has focused on showing an inverse relationship only for written monolog, only for frequencies and ranks of one linguistic unit, generally word unigrams, with strong correlations of the power law to the observed frequency distributions, with limited to no attention to psychological mechanisms such as the principle of least effort. The current paper extends the existing findings, by not focusing on written monolog but on a more fundamental form of communication, spoken dialog, by not only investigating word unigrams but also units quantified on syntactic, pragmatic, utterance, and nonverbal communicative levels by showing that the adequacy of Zipf’s formula seems ubiquitous, but the exponent of the power law curve is not, and by placing these findings in the context of Zipf’s principle of least effort through redefining effort in terms of cognitive resources available for communication. Our findings show that Zipf’s law also applies to a more natural form of communication—that of spoken dialog, that it applies to a range of linguistic units beyond word unigrams, that the general good fit of Zipf’s law needs to be revisited in light of the parameters of the formula, and that the principle of least effort is a useful theoretical framework for the findings of Zipf’s law.

**Supplementary Information:**

The online version contains supplementary material available at 10.3758/s13423-022-02142-9.

## Introduction

One might expect word frequencies across corpora to be variable, as language users have full flexibility and control over their word use and the frequency with which they use those words. Yet a relatively simple mathematical formula shows that variations of word frequency distributions are in fact very much constrained. Estoup (1912) reported this regularity, now commonly known as Zipf’s law, after the linguist Georg K. Zipf (1932, 1935) who popularized it. Zipf’s law states that given a vocabulary of word types, the frequency of occurrence *f* of a word type is inversely correlated with the rank *r* of that word type in an ordered frequency distribution, as shown in Equation 1. In this equation, *C* is a constant, which can be calculated using the Hurwitz Zeta function^1^, *r* is the rank of a word type, and *α* the value of the exponent characterizing the distribution. Typically, *α* is assumed to be approximately 1, but deviations have been reported in the literature (see, e.g., Ferrer-i-Cancho, 2005; Mehri & Jamaati, 2017; Zipf, 1949).

1 $$f\left(r;\alpha, C\right)=\frac{C}{r^{\alpha }}$$

Some have argued that the elegance of simplicity of Zipf’s law is in fact an oversimplification because word frequency distributions are more complex and cannot be captured in a mathematically simple formula (Mandelbrot, 1953; Piantadosi, 2014). As a consequence, variations to Zipf’s law have been proposed, the most common one being introduced by Mandelbrot (1953) to account for the true frequency distribution of words. The frequency of observation *f* of the word type at rank *r* can be calculated using Equation 2. The fit of the Zipf–Mandelbrot formula is generally better than that of Zipf’s formula. This is not surprising as the formula is very similar to Zipf’s law, yet contains the additional parameter *β*.

2 $$f\left(r;\alpha, \beta, C\right)=\frac{C}{{\left(r+\beta \right)}^{\alpha }}$$

There are four reasons for the current paper to focus on Zipf’s original formula rather than alternatives like the Zipf–Mandelbrot formula. First, when explaining possible mechanisms behind a regularity, it is considerably easier to deal with two rather than three parameters, in line with the principle of parsimony. Second, because the Zipf–Mandelbrot formula has an additional parameter, it can capture a larger variety of distributions. Consequently, the findings for the Zipf formula can automatically be extended to the Zipf–Mandelbrot formula, but not vice versa. Third, for the original formulation of Zipf’s law, theoretical predictions have linked the exponent to psychological mechanisms (Baixeries et al., 2013; Ferrer-i-Cancho, 2005). Finally, if we aim to better understand the cognitive implications of Zipf’s law, most notably the ones Zipf pointed out himself, using Zipf’s law as a starting point is preferred over a derivation of the original formula. A comparison of the performance of variations of Zipf’s law, including Zipf’s original formula and the Zipf–Mandelbrot formula is undoubtedly important (cf. Baayen, 2001; Moreno-Sánchez et al., 2016), but given that the focus of the current paper is less about goodness of fit and more about mechanisms, we here focus on the source of these variations of Zipf’s law, Zipf’s original formula as defined in Equation 1.

A large number of studies have investigated Zipf’s law in the context of language (e.g., Ferrer-i-Cancho, 2005; Ha et al., 2002; Li, 1992; Piantadosi, 2014; Zipf, 1935, 1949). Most research has focused on showing an inverse relationship for the frequency of a word and its rank, primarily in written monolog. Most research has restricted the variables used to test Zipf’s law to one linguistic unit, primarily word unigrams. The rank-frequency relation of unigrams in these studies have generally focused on the strong correlations of the power law to the observed frequency distributions. Meanwhile, these studies have paid no (or very little) attention to the psychological mechanisms that Zipf pointed out, the *principle of least effort* (Zipf, 1949). Each of these items—spoken dialog, linguistic units, goodness of fit, and principle of least effort—will be discussed next.

### Spoken dialog

Zipf’s law was originally shown for written language (Zipf, 1932, 1935). The word frequencies of American newspapers, Latin of Plautus, and Chinese of Peiping—to include only some corpora Zipf (1935) used—all followed Zipf’s law. Many studies have been conducted showing that Zipf’s law applies to virtually any written text in any language, thereby focusing on monolog (Ha et al., 2002; Németh & Zainkó, 2002; Piantadosi, 2014). The generalizability of Zipf’s law for different text corpora made Zipf speak about a *psychobiology of language* (Zipf, 1935). The irony, however, is that written monolog is not the most natural form of language. Though it is explainable why Zipf applied his formula to written monolog—for the same reason corpus linguistics generally uses written text—evidence for written monolog does not necessarily yield evidence for a more natural form of language, that of spoken dialog.

If Zipf’s law is a regularity in human language communication, a psychobiology of language, then it must at least apply to the most natural form of human language communication, that of spoken dialog. After all, spoken dialog is the first mode of communication we acquire (i.e., before written communication), it is acquired by most of us without any instruction (contrary to written communication), it precedes written communication in language evolution and it is more adaptive in the language evolutionary process compared with written language (Clark, 1996). Importantly, in many respects spoken dialog has very different features compared with written monolog. Spoken dialog has been described as a joint action of language use (Clark, 1996). It can be characterized by the fact that participants share the same physical environment (co-presence), participants can see each other (visibility), participants can hear each other (audibility), participants perceive each other’s actions at no perceptible delay (instantaneity), the medium fades quickly (evanescence), participants’ actions leave no record or artifact (recordless), participants can produce and receive at once and simultaneously (simultaneity), participants formulate and execute their actions real time (extemporaneity), participants determine for themselves what actions to take when (self-determination), and participants take actions as themselves (self-expression; Clark & Brennan, 1991). Unlike any form of written language, spoken dialog requires both the activation of the speech comprehension and speech planning systems within very short time windows, imposing significant cognitive load on the participants (Pickering & Garrod, 2004, 2013). It has also been argued that participants synchronize different channels to alleviate cognitive load and enhance common ground (Louwerse et al., 2012; Pickering & Garrod, 2004, 2013).

The number of studies that have put Zipf’s law to the test for spoken dialog is limited, especially compared with the vast number of studies that make use of written monolog. There are some notable exceptions (Baixeries et al., 2013; Bian et al., 2016; Hernández-Fernández et al., 2019; Lin et al., 2015; Neophytou et al., 2017;Ridley, 1982 ; Torre et al., 2019). However, these exceptions generally make use of a small language corpus of data (Ridley, 1982; Torre et al., 2019), focus on a specific group of speakers (Baixeries et al., 2013; Neophytou et al., 2017), or use heterogenous data where speech was taken from a variety of settings (Bian et al., 2016; Hernández-Fernández et al., 2019; Lin et al., 2015). To our knowledge no comprehensive study has investigated the extent of Zipf’s law with regards to the most natural form of communication, spoken dialog, and has included the various aspects that make dialog so different from monolog, and spoken language so different from written language.

### Linguistic units

The vast majority of the research on Zipf’s law within language has focused on word unigrams (Piantadosi, 2014). There are, however, some exceptions of studies that have looked at other linguistic units. Good fits to Zipf’s formula (or one of its alternative formulations) have been found for part-of-speech (PoS) tag frequency distributions (Piantadosi, 2014; Tuzzi et al., 2010), higher order *n*-grams sizes beyond word unigrams (Ha et al., 2009) and the frequency distribution of number words (Dehaene & Mehler, 1992). More recently, some studies have focused on the more physical dimension of speech sounds and sequences, rather than their symbolic representation (Baumann et al., 2021; Torre et al., 2019). These studies have argued that quantitative linguistic laws, including Zipf’s law have a physical origin, rather than only applying to written text. Baumann et al. (2021) investigated Zipf’s law in sequences of phonemes within and across words in a dialog corpus and found high quality fits with the law, especially for longer sequences. These studies are extremely insightful, yet they do not shed light on the multitude of aspects that are so characteristic to dialog, such as the utterance length, the utterance beginning or end, the (simplified) syntax and the dialog act, in addition to linguistic units such as word unigrams and bigrams.

### Goodness of fit

Typically, Zipf’s law (but also the Zipf–Mandelbrot formula) has been fitted by estimating its parameters using the maximum likelihood estimate (MLE). MLE has been shown to give better fits than its alternative, a linear regression on a log-log plot. The linear regression on a log-log plot has been argued to produce biased and inaccurate fits (Goldstein et al., 2004). The resulting *R*^2^ determination coefficient quantifies the variance that the fit of the Zipf (and Zipf–Mandelbrot) formula can explain for each frequency distribution (Piantadosi, 2014; Ridley, 1982; Tuzzi et al., 2010). There is however no set standard how low or how high the *R*^2^ determination coefficient should be in order for Zipf’s law to apply. Studies on Zipf’s law typically report very high *R*^2^ values of above .8 (Mehri & Jamaati, 2017; Piantadosi, 2014; Ridley, 1982; Tuzzi et al., 2010).

There may be better alternatives to measure the quality of the fit, for example through the Kolmogorov–Smirnov statistic (Clauset et al., 2009). However, the Kolmogorov–Smirnov statistic is not without problems either (Geller, 1979), as it is very restrictive in accepting Zipf-like distributions. Piantadosi (2014) added, “In general, it is not so important which simple distributional form is a better approximation to human language. What matters more are the general properties of word frequencies that are informative about the underlying mechanisms behind the observed distribution” (p. 1116). Perhaps most importantly, the far majority of studies report the *R*^2^ determination coefficient as a goodness of fit (e.g., Baumann et al., 2021; Mehri & Jamaati, 2017; Neophytou et al., 2017; Piantadosi, 2014; Ridley, 1982; Tuzzi et al., 2010). Since we are primarily interested in studying the underlying mechanisms of Zipf’s law rather than studying the maximum goodness of fit, we intentionally steer away from a debate on when a distribution is such that Zipf’s law applies. Instead, we use the *R*^2^ determination coefficient as a diagnostic tool only, as it is most intuitive and is the one most commonly used (see, e.g., Baumann et al., 2021; Mehri & Jamaati, 2017; Neophytou et al., 2017; Piantadosi, 2014; Ridley, 1982; Tuzzi et al., 2010).

Studies reporting the goodness of fit for Zipf’s law (or its derivative Zipf–Mandelbrot) have typically reported *R*^2^ values above .8 (Mehri & Jamaati, 2017; Piantadosi, 2014; Ridley, 1982; Tuzzi et al., 2010), and adjusted *R*^2^ values of .98 or higher (Piantadosi, 2014). With such high values, these findings suggest that Zipf’s law is ubiquitous, perhaps so much so that Zipf’s law says little about language or the psychobiology of language, and merely is a statistical phenomenon. Indeed, Zipf’s law has also been observed in many other nonlanguage domains. As early as 1913, it was noted that the distribution of city population sizes follows a similar regularity (Auerbach, 1913). But Zipf’s law has also been found for web page visits (Adamic & Huberman, 2002), pitch patterns in contemporary Western music (Serrà et al., 2012) and the (ordered) frequency distribution of different opening moves in chess (Blasius & Tönjes, 2009; see Būdienė & Gruodis, 2016, for an overview). Even (simulated) monkeys that type letters on a keyboard have produced texts where the letter strings follow Zipf’s law (Li, 1992; G. A. Miller, 1957). However, these findings have been contested with evidence showing that the frequency distributions from random texts are statistically different from distributions from natural language (Ferrer-i-Cancho & Elvevåg, 2010; Ferrer-i-Cancho & Gavaldà, 2009).

More importantly for the current paper is that the high goodness of fit values might obfuscate important information that makes Zipf’s law more than simply a statistical explanation. We illustrate this point here by constructing a fictitious corpus of 13,900 (nonsense) items. The most frequent item, ranked first, has a hypothetical frequency of 10,000, the words ranked 2–4 each have a frequency of 1,000, the words ranked 5–7 have a frequency of 200, and those ranked 8–10 a frequency of 100. We purposefully keep the word distribution extremely simple. When applying Zipf’s law using the equation presented earlier, the *R*^2^ is notoriously high at .98, a goodness of fit that has often been found for fitting Zipf’s law, even when considering different exponents that have been found for different languages (Mehri & Jamaati, 2017) or different groups of individuals (Baixeries et al., 2013; Hernández-Fernández & Diéguez-Vide, 2013; Neophytou et al., 2017). The *C* value is a constant that is dependent on the sample size and is generally not of particular interest. However, the *α* value of 2.13 very much differs from the *α* values commonly observed in language, which tend to lie in the range of 0.6 and 1.5 (Ferrer-i-Cancho, 2005). This is an important observation as it shows that even though the *R*^2^ may show a near perfect fit with the data, it yet hides the values of the underlying variables that may turn out to be more informative. Moreover, as we will see later, it is not the *R*^2^ but the *α* value that reveals the psychological mechanisms behind Zipf’s law, the so-called principle of least effort.

### Principle of least effort

Piantadosi (2014) aptly stated that “unfortunately, essentially all of the work in language research has focused solely on deriving the law itself in principle; very little work has attempted to assess the underlying assumptions of the hypothesized explanation” (p. 1113). Whereas Zipf (1932) demonstrated evidence for the regularity in language, Zipf (1949) provided an explanation for the regularity, the principle of least effort. According to this principle, humans always try to optimize their behavior by minimizing their effort to get to the goal they are trying to achieve. Zipf argued that speakers try to minimize effort by using the smallest vocabulary of words as possible, while hearers prefer less ambiguity in a word use and hence a larger vocabulary to express all possible concepts.

Stemming from the same principle of least effort, language users use shorter words more frequently than longer words (Piantadosi et al., 2011; Zipf, 1949), a law often referred to as *Zipf’s law of abbreviation*. Zipf (1949) explained this phenomenon from an evolutionary perspective by arguing that more frequently used words would benefit more from a reduction in their size and this, in turn, would lead to more efficient communication. Speakers economize their efforts by using a force of unification, using a few different words but each of them frequently. Hearers on the other hand economize their efforts using a force of diversification—preferring many different words in their communication, each of them being relatively infrequent.

For the principle of least effort and the forces of unification and diversification to apply, communication between speaker and hearer is needed. The lion’s share of evidence supporting Zipf’s law, however, comes from communication in which the speaker at best assumes a role of the hearer, as is the case in written monolog. In those cases of written monolog, perhaps the speaker takes into account the hearer economizing her effort using the force of diversification, but this is a presumed hearer. Instead, for the principle of least effort and the subsequent forces to be validated, natural communication needs to be taken into account.

For written monolog, it is unlikely that speakers try to economize linguistic effort in the same way and to the same extent as in dialog, especially in spoken dialog, as they need to maximize information to an unknown listener. In spoken dialog, however, speakers generally find their hearers being present (Clark, 1996; Goodwin, 1981; Schegloff, 1996). They know whether they have the hearer’s attention, whom they are talking to, when they can start and stop speaking, and what they can say. Hearers often give cues by providing feedback. Overall, speakers and hearers coordinate their action by monitoring whether hearers are attending to what is said, who they are talking to, when they are speaking, what to say, and whether the speaker needs to follow up on an earlier piece of information. Furthermore, spoken conversations take place in a specific environment and context, which is reflected in the language use. The language used by someone in a conversation with colleagues will be substantially different from the language that the same person used in a conversation with family members, for example. All these factors make spoken dialog a very dynamic act of coordination (Clark, 1996; Louwerse et al., 2012; Louwerse & Mitchell, 2003). Importantly, spoken dialog can be considered a more natural form of communication than written monolog or dialog (Clark, 1996). This means that in order for the principle of least effort to be true, for a cognitive origin of an explanation for Zipf’s law being found in natural language, we must at least be able to find it in a more natural, more spontaneous, more intentional and a more dynamic register than written language, that of spoken dialog. Moreover, we can expect that if the context of the dialog places a higher cognitive load on the speaker—for instance because of a cognitive task—the speaker will have to economize linguistic effort even further due to these extralinguistic cognitive constraints.

It is worth mentioning that the principle of least effort itself does not make any predictions about how the degree of economization of linguistic effort influences the parameters of Zipf’s law. Moreover, Zipf applied the principle beyond language and cognition, and applied it to other domains, such as city populations and economics (Zipf, 1949). But because the principle does assume that the result of the economization of linguistic effort yields Zipf’s law, the effects of linguistic effort ought to be measurable within the distribution. More in particular, the effects should be measurable in either one of the two parameters that characterize a Zipfian distribution: the steepness of the slope, measured through *α* or the goodness of fit, measured through the *R*^2^ value (the constant not being a useful candidate as it is dependent on the sample size). Most studies on Zipf’s law report the (high) goodness of fit values (Piantadosi, 2014), but as we argued earlier these values obfuscate underlying patterns. For those studies that reported *α* values, interesting patterns can be observed. Lin et al. (2015) observed slightly higher *α* values for spoken language than for written language. Others have reported that *α* values decreased with age in the speech of young children in interaction with adults (Baixeries et al., 2013). Yet other studies showed that patients with language deficits or cognitive impairments have deviating *α* values compared with control groups (Hernández-Fernández & Diéguez-Vide, 2013; Neophytou et al., 2017). Because the economization of the speaker’s language is captured in the force of unification, higher *α* values can be expected as a consequence of a decrease in word types. We may therefore expect that an increase in cognitive load results in more economization on the speaker’s side, yielding higher *α* values.

**Fig. 1:**
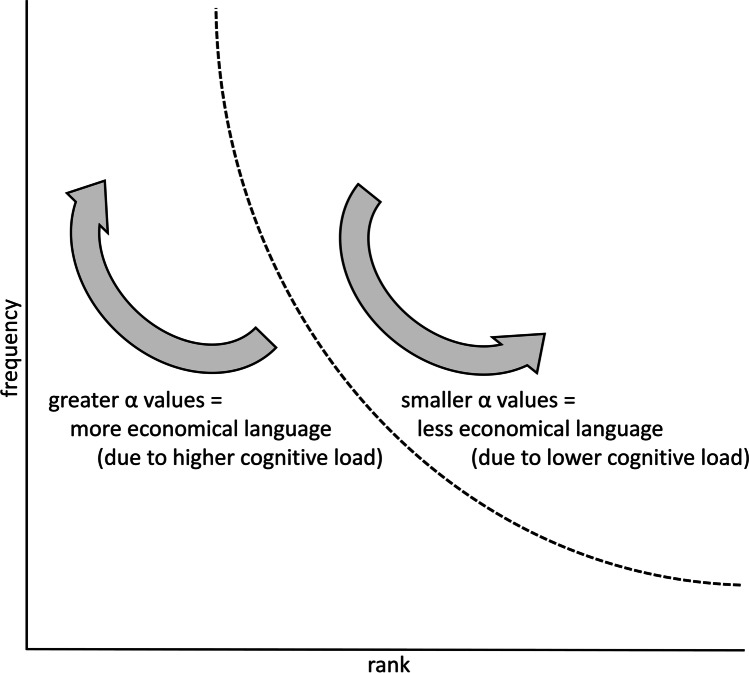
Illustration of the expected behavior of the influence of cognitive effort on the steepness of the slope of a Zipfian curve. The steepness of the slope of the curve, quantified by α, representing Zipf’s law (dotted line) is expected to increase with more economical language, due to a higher cognitive load on a task and is expected to decrease with less economical language, due to a lower cognitive load on a task

Based on the principle of least effort and the forces of unification and diversification, we hypothesized that the steepness of the slope, as determined by the *α* value, correlates with the economical linguistic contribution, as illustrated in Fig. 1. That is, we propose a *cognitive effort hypothesis* that predicts that if the cognitive load of an extralinguistic task increases, language users will be more economical in their linguistic contributions. More specifically, if more cognitive effort is required, language users will rely on using fewer words with higher frequency. Conversely, if there are more cognitive resources available, language users will not economize on the communicative task at hand and will use more different words each having a lower frequency. Consequently, with a task requiring more cognitive effort, more economical language needs to be used, and the slope of the Zipfian curve will become steeper, as demonstrated by higher values for *α*. With a task requiring less cognitive effort, less economical language needs to be used yielding a Zipfian curve that is not as steep, as demonstrated by lower values for *α*. Note that the cognitive effort hypothesis predicts an effect for the *α* value (the steepness of the curve) and not necessarily the *R*^2^ (the general fit of the formula).

So far we have discussed Zipf’s law, the importance of considering the most natural form of language, the different linguistic units reported in the literature, the limitations of the commonly reported goodness of fit and the explanation for Zipf’s law in the principle of least effort. We broke the rest of the paper into two parts. In the first part we focused on Zipf’s law. First, we measured the extent to which Zipf’s law is present in dialog by using spoken dialog corpora on a wide variety of dimensions, such as corpora that differed in the language being used, whether the conversation took place face-to-face or over the phone, whether the participants knew each other, and whether the dialog was task- based or not. Second, we investigated the extent to which Zipf’s law extends beyond word frequencies by considering larger units, such as word bigrams and whole utterances and words at specific positions in the utterance. Furthermore, we extended the analysis to the level of syntax, pragmatics and nonverbal communication channels. In the second part of the paper, we investigated the extent to which these findings can be explained by the principle of least effort through investigating the validity of the proposed cognitive effort hypothesis.

## Zipf’s law in spoken dialog

### Corpora

Albeit more natural than any form of written language and therefore even more worthwhile to take into account than written monolog, spoken human-human dialog corpora are also more difficult to obtain than written language corpora. This is no surprise, as spoken human-human dialog is evanescent and recordless and requires the additional effort of having to be transcribed to be used for many computational linguistic analyses. Dialog corpora containing annotations of nonverbal behavior are even scarcer. In addition to transcriptions, they require careful annotations preferably conducted by multiple annotators.

Despite their sparsity, we gathered a collection of corpora with transcribed speech and other communicative units from natural conversations. Natural conversations take place in a wide variety of settings and between a wide variety of participants. We tried to approximate this diversity but also tried to allow for generalizing the results as much as possible by identifying groups of corpora. A number of criteria determined the selection: (1) Each corpus should comprise spoken interactions between at least two participants in a dialog setting, (2) each participant should have roughly an equal opportunity to take the floor (3) with each corpus containing at least 20 dialogs, and (4) with all content of a corpus being created using similar steps, conditions and goals in mind as outlined below. Importantly, given the scarcity of corpora we created subsets by splitting each corpus in different samples, ensuring an equal (minimal) sample size. Corpora were clustered by free-flow situated conversations, free-flow telephone conversations, task-based conversations, and dialog with nonverbal annotations.

Before explaining the corpora in more detail, we summarize the similarity and differences between the corpora in Table 1. This table furthermore contains frequency statistics on the number of dialogs, utterances and word tokens and types. In total we used 28 corpora, two of which were annotated with nonverbal information. The corpora have been clustered, based on the type of dialog they contain. All corpora were divided into utterances where full conversational turns, bounded by the speech of one or more other dialog participants, can consist of multiple utterances.

**Table 1:** Overview of the corpora and their properties and frequency statistics

Dialog Type	Corpus	Language	Face-to-face	Familiar	Task-based	Strict roles	Adults	Dialogs	Utterances	Tokens	Types
Mixed	Santa Barbara Corpus	English	±	±	±	±	±	60	40,539	249,858	12,316
Face-to-Face	Croatian Spoken Language Corpus	Croatian	+	+	−	−	+	164	59,039	288,480	26,823
	Griffith Corpus of Spoken Australian English	English	+	+	−	−	+	40	4,710	38,172	4,257
	Spoken Dutch Corpus										
	Face-to-Face Section	Dutch	+	+	−	−	+	1,537	392,607	2,572,002	58,457
	ORAL2013 Corpus	Czech	+	+	−	−	+	835	512,585	2,785,189	127,857
	Garvey Corpus	English	+	+	−	−	−	46	10,027	49,484	2,206
Telephone	Spoken Dutch Corpus										
	Telephone Section	Dutch	−	+	−	−	+	1,230	307,106	2,052,707	44,687
	Switchboard Corpus	English	−	−	−	−	+	1,155	216,747	1,453,753	21,721
	CallFriend										
	English	English	−	+	−	−	+	41	21,058	122,465	7,839
	Farsi	Farsi	−	+	−	−	+	100	32,240	234,364	17,420
	Japanese	Japanese	−	+	−	−	+	32	31,307	161,760	10,098
	Korean	Korean	−	+	−	−	+	100	40,828	225,510	30,333
	Spanish	Spanish	−	+	−	−	+	124	84,630	581,577	23,695
	CallHome										
	Arabic	Arabic	−	+	−	−	+	141	31,421	196,035	21,719
	English	English	−	+	−	−	+	176	34,706	243,299	9,855
	German	German	−	+	−	−	+	120	32,000	215,402	13,723
	Japanese	Japanese	−	+	−	−	+	120	39,390	199,048	12,145
	Mandarin	Mandarin	−	+	−	−	+	140	38,724	240,774	9,047
	Spanish	Spanish	−	+	−	−	+	140	31,196	237,674	13,223
Task-based	AMI Meeting Corpus	English	+	+	+	−	+	171	128,038	978,215	14,376
	ICSI Meeting Corpus	English	+	+	+	−	+	75	108,652	833,054	12,085
	HCRC Map Task Corpus										
	Eye Contact and Familiarity	English	+	+	+	+	+	32	7,021	39,541	1,444
	Eye Contact and No Familiarity	English	+	−	+	+	+	32	5,253	30,797	1,201
	No Eye Contact and Familiarity	English	−	+	+	+	+	32	7,939	43,591	1,424
	No Eye Contact and No Familiarity	English	−	−	+	+	+	32	6,871	39,851	1,291
	TRAINS93 Corpus	English	−	−	+	+	+	98	7,020	55,785	1,131
	Walking Around Corpus	English	−	−	+	+	+	36	19,847	201,027	3,851
Nonverbal	Memphis Multimodal Map Task Corpus	English	+	−	+	+	+	192			

+ and − refer to the presence and absence of that dimension in the corpus, respectively. When a corpus consists of conversations where a dimension is both present and absent, it is indicated by ±

Firstly, the Santa Barbara Corpus of Spoken American English was included (Du Bois et al., 2000). This corpus contains primarily free-flow situated conversations recorded in a wide variety of informal and private, and situated and non-situated settings across the United States. This corpus is not homogenous as it also includes dialog recorded in formal, task-based and non-situated settings. It is included for reasons of completeness.

#### Free-flow face-to-face conversations

We included five general-purpose corpora containing free-flow, face-to-face conversations. Four of these corpora have a similar setup and were designed with the goal of creating a representative collection of everyday dialog in a natural, informal and familiar setting. These corpora are the Croatian Spoken Language Corpus (Kuvač Kraljević & Hržica, 2016), the Griffith Corpus of Spoken Australian English (Haugh & Chang, 2013), the Spoken Dutch Corpus (Oostdijk, 2000) and the ORAL2013 Corpus of informal spoken Czech (Benešová et al., 2015). Except for the Griffith Corpus of Spoken Australian English, the corpora are fairly large and all four corpora represent a wide variety of speakers from different regions and dialects. For the Spoken Dutch Corpus, there is a significant portion of the speakers from Belgium, speaking the Flemish dialect.^2^ In addition, a corpus of child speech was used (Garvey, 1979). This corpus contains interactions of children between 2 and 6 years of age. With children generally having a smaller vocabulary size, their frequency distributions and the resulting parameters of Zipf’s law can be expected to differ from those of adults (Baixeries et al., 2013; Segbers & Schroeder, 2017).

#### Free-flow telephone conversations

The dynamics of free-flow situated conversations are different than for instance telephone conversations (Doherty-Sneddon et al., 1997; Ten Bosch et al., 2004). So, in addition to situated interactions, we included a corpus of telephone conversations between participants who were familiar with each other, the Spoken Dutch Corpus (telephone section; Oostdijk, 2000), and a corpus of telephone conversations between two strangers about a topic of choice, the Switchboard Corpus (Godfrey et al., 1992). Similarly, we included two series of corpora containing telephone conversations between acquaintances: CallFriend in the languages American English, Farsi, Japanese, Korean, and Spanish and CallHome in the languages Arabic, American English, German, Japanese, Mandarin, and Spanish. The construction and annotation of the CallFriend and CallHome corpora in the different languages were very similar, which allowed for a more structural comparison of Zipf’s law across languages. In all the corpora, the calls were initiated by someone in the United States, and the caller was free to choose whom to call and where to call to, and hence generally chose to call a close family member or friend. This means that both participants in each dialog knew each other well. The CallFriend and CallHome corpora were retrieved from the Talkbank data collection initiative (MacWhinney, 2007), except for the CallFriend Farsi and Korean corpora, which were retrieved from the Linguistic Data Consortium (Ko et al., 2003; D. Miller et al., 2014). Note that familiarity between the speakers might also influence the dynamics of conversations (Bard et al., 2002; Branigan et al., 1999).

#### Task-based conversations

In task-based dialog, naturally the interactions between participants are very different from those taking place in a more spontaneous setting. We included two corpora with dialogs, recorded at meetings. Contrary to all other corpora included in this study, these dialogs consisted of more than two participants. The AMI Meeting Corpus contains both dialogs of real meetings and dialogs of simulated meetings with participants playing a particular role (Carletta et al., 2005). The real meetings took place in a variety of settings, while the simulated dialogs took place in a fictional electronics company and were about a product design. Dialogs had four or five participants. The ICSI Meeting Corpus contains dialogs, recorded at the International Computer Science Institute, which were mainly about the creation of the corpus itself (Janin et al., 2003). Each dialog had between four and ten participants. While the participants in these corpora can be said to have a specific role, especially in the AMI Meeting Corpus, we did not annotate these corpora as having strict roles, because we hypothesized that the role of the speaker would not have a significantly large impact on the conversation, in contrast to the task-based corpora below, where the speakers were arguably more restricted in their linguistic freedom than in the meetings corpora, due the role they had in the task.

In addition to the corpora with meeting dialogs, we included a set of corpora, where the conversations had a clearly defined goal that the participants collaboratively worked toward. The HCRC Map Task Corpus contains dialogs between two participants that had to collaboratively solve a road map (Anderson et al., 1991). Both participants got a slightly different map and could not see the map of the other. This was done, so that an interaction between the participants was necessary to solve the task. In each dialog, there was a clear distinction between the roles of the participants, with an instruction giver providing the route map instructions and an instruction follower recreating the route using the instructions. The corpus had a 2 × 2 design, with half of the participant pairs not being able to make eye contact and half of the participant pairs knowing each other well, prior to the dialog. Since we annotated each corpus for familiarity and the possibility to make eye contact, we split this corpus into four separate parts, each containing an equal number of dialogs. The TRAINS93 Corpus contains dialogs between two participants who had to collaboratively solve a freight planning task, with one participant playing the “user,” who had to solve the task and the other participant playing the “system,” who had to assist the user, by providing information that helped the user to successfully come up with a solution (Heeman & Allen, 1995). The goal of the corpus was to acquire dialogs that could be used to build an automated planning assistant. Again, one can easily distinguish two different speaker roles in this corpus. Finally, the Walking Around Corpus had a similar setup to the Map Task scenario, but in this case, an instruction follower had to physically walk the route to different destinations on a university campus, using the instructions received through telephone from an instruction giver (Brennan et al., 2013).

#### Dialog with nonverbal annotations

Two corpora with multimodal annotations were included. The AMI Meeting Corpus contains annotations on head gestures for a substantial number of the dialogs. We also included the Memphis Multimodal Map Task Corpus (Louwerse et al., 2012). This corpus has a very similar setup as the HCRC Map Task Corpus. What differentiates this corpus from the HCRC Map Task Corpus, is that it focused on annotating multimodal information. The corpus contains annotations of gestures, facial expressions and linguistic information, such as dialog acts, landmark descriptions, and discourse markers. Unlike in the corpora described so far, the behavioral events (e.g., gestures, dialog acts, discourse markers) were annotated in a time series through binary encoding, while the transcriptions were not. In the current study, we only considered the nonverbal annotations of this corpus.

### Corpus preprocessing

We preprocessed all corpora to a minimal extent by removing punctuation marks and annotation comments and characters, so that only the actual speech was used in the analysis. Note that in speech, words can be uttered with slightly different pronunciations, intensities, and duration, which could alter their meaning. Such variations, however, are not kept in most transcriptions. If they were present, we removed them as they were part of the annotations. We kept the division into turns and utterances the way it was annotated, even though annotators across corpora might have used different criteria for dividing dialog into turns and turns into utterances.

### Linguistic units

We included a number of different linguistic units into our analysis. These included units on word, utterance, syntactic, and pragmatic levels.

#### Word level

Most studies on Zipf’s law in language have focused on word unigram frequencies. Next to word unigram frequencies, we also investigated larger units than individual words, more specifically, word bigrams (Ha et al., 2002; Ha et al., 2009). Finally, more important in dialog than in written monolog are the first and last words in an utterance or turn. At least for English, the words in these positions are more often used to connect utterances or turns (Louwerse & Mitchell, 2003). We therefore investigated specifically the presence of Zipf’s law in frequency distributions of the first and last words of an utterance.

#### Utterance level

Williams et al. (2015) suggested that Zipf’s law occurs at the level of phrases. Since spoken dialog is very dynamic, turn and utterance segments are generally short (Levinson & Torreira, 2015). Because these segments are short and likely repeated, we expected the frequency distribution over utterances to be nonuniform. Therefore, we included the frequency distribution over utterances, where each unique utterance is taken as an individual countable observation. Frequency distributions over the lengths of words follow Zipf’s law of abbreviation with shorter word occurring more frequently (Piantadosi et al., 2011; Zipf, 1949). The tendency to reduce the length of words can be explained by the principle of least effort (Ferrer-i-Cancho et al., 2022; Zipf, 1949). Since this tendency likely appears at many levels in language and communication, we hypothesized that Zipf’s law of abbreviation also applies to the level of utterance lengths, with an inverse relationship between the length of an utterance as measured in terms of number of words and its frequency of occurrence. Indeed, we found this to be true on a small scale in dialogs between a dialog system and a user (Linders & Louwerse, 2020). Here, we extended the findings to larger scale by considering a larger and more varied selection of corpora. However, we would like to point out that Zipf’s law of abbreviation can likely be better approximated with a geometric distribution than with Zipf’s formula (Ferrer-i-Cancho et al., 2022). Nonetheless, we approximated the distribution with Zipf’s formula in the current paper for two reasons. First, we are not interested in the most optimal fit and for the sake of keeping the analysis simple, we preferred approximating the distribution using the same formula. And second, it is likely that the same principle of least effort applies to both Zipf’s law and Zipf’s law of abbreviation (Ferrer-i-Cancho et al., 2022; Zipf, 1949). Also note that we are not plotting the frequency against the rank of the word, but against the “size” of the observation. Hence, the observations are not ordered on frequency, but on the length of the observation itself, essentially substituting this variable with the rank.

#### Syntactic level

Part-of-speech (PoS) tag frequencies have also been approximated using Zipf’s law (Piantadosi, 2014; Tuzzi et al., 2010). However, Tuzzi et al. (2010) also showed that PoS tag distributions are better approximated by an exponential distribution than by Zipf’s formula. Likewise, Ferrer-i-Cancho et al. (2022) provided mathematical arguments why a geometric distribution is a better fit to PoS tag unigram distributions. Again, for the same arguments as presented for the utterance length unit, we proceeded with approximating these distributions using Zipf’s formula. Both the Spoken Dutch Corpus and HCRC Map Task Corpus include PoS tags, which were automatically annotated and manually corrected. Using the Stanford Neural Pipeline (Qi et al., 2020), we automatically tagged all English corpora, except for the two Map Task Corpora. The Stanford Neural Pipeline was trained on the Universal Dependencies English Web Treebank and makes use of the Universal PoS tagset (Petrov et al., 2012). The Stanford Neural Pipeline in combination with a model trained on the Universal PoS tagset was chosen because the tool is accessible yet has a high tagging accuracy of around 95% (Qi et al., 2020), and the tagset was constructed to be agnostic to the language and domain of the corpus (Petrov et al., 2012). Because of the high accuracy, the automatic PoS tags can be used reliably in the analysis. The tagset of the Spoken Dutch Corpus consists of 11 tags, the HCRC Map Task Corpus of 58 tags and the Universal Dependencies tagset of 17 tags. In our analysis, we included both PoS tag unigram frequency distributions, as well as frequency distributions of two consecutive PoS tags: PoS bigrams. Analogous to the word level units, we included the frequency distribution over the first and last PoS tags of an utterance in our analysis and similar to the utterance level units, we also included the frequency distribution of the PoS sequences of utterances. Finally, somewhat analogous to the utterance length unit, we divided the PoS tagsets into lexical and grammatical tags, based on Universal Dependencies distinction of “open” and “closed” classes, and counted the number of lexical and grammatical tags in an utterance.

#### Pragmatic level

In addition to word, utterance, and syntactic levels, Zipf’s law can also be investigated on a pragmatic level. To quantify this, we looked at speaker intentions in dialogs. Speaker intentions are often annotated, usually on an utterance level, with speech or dialog acts capturing the illocutionary act of the speaker (Austin, 1962; Searle, 1976). Five corpora in our analysis contain dialog act annotations. The Switchboard Corpus contains 220 multidimensional dialog acts, but the common practice is to cluster them to a set of 42 dialog acts (Jurafsky et al., 1997). In our analysis we made use of the clustered set. The AMI Meeting Corpus was annotated with a set of 15 dialog acts. The ICSI Meeting Corpus contains annotations, using a tagset of 56 tags. The dialog acts could be analyzed in four different manners. Tags could be divided into general and specific tags and an utterance could have multiple general and specific tags. There are 11 general tags and 41 specific tags.^3^ In addition, we could split all the tags and analyze the frequencies of each of these tags (which includes two additional tags, compared with the general and specific tags marking turn abandonment and interruptions). Finally, for each utterance, one could take all the full labels, without separating them into individual tags, leading to 2,052 unique tags. The HCRC Map Task Corpus and Memphis Multimodal Map Task Corpus have been annotated with the same tagset of 13 tags, including an “uncodable” label.

#### Nonverbal level

Nonverbal behavior is a significant part of face-to-face dialog (Clark, 1996). Since much of facial expressions and gestures are communicative in nature, we might expect Zipf’s law and the least effort principle to apply to nonverbal communication as well. Yet, there is no research on whether nonverbal behavior channels in human dialog also follow Zipf’s law. There is however some evidence from animal communication, with gorilla gestures following Zipf’s law (Genty & Byrne, 2009).

For the AMI Meeting Corpus, we analyzed two nonverbal channels: hand and head gestures. Both hand and head gestures were annotated for their communicative function and do not follow an established annotation scheme and hence are custom. In both channels, we removed the tags indicating the speaker used a noncommunicative signal or was off-camera. In total, there were seven tags for head gestures and 13 for hand gestures. Ten of these are deictic gestures pointing toward persons (one tag for each of the four speaker roles), objects or a location.

Next to dialog acts, the Memphis Multimodal Map Task Corpus was annotated for gestures, face touches and eye, eye brow, mouth and head movements. To be consistent with Louwerse et al. (2012), we kept their taxonomy and treated gestures as a group and eye and facial movements as a group. Because the group of face touches only contained two categories (analogous to two words in a written corpus), we excluded this group from the analysis. Gestures were identified and classified according to McNeill’s (1992) coding system, which contained beat, deictic, iconic, metaphoric, and symbolic gestures (Louwerse & Bangerter, 2010). Louwerse et al. (2012) classified the beat gestures further into single and multiple beats, deictic gestures into concrete and abstract variants, iconic gestures into those referring to landmarks and those referring to the route and metaphoric gestures into those referring to a meta-action and those referring to an action. Symbolic gestures did not occur in the data. The encoding of eye and facial movements was largely based on the Facial Action Coding System (FACS) by Ekman et al. (2002), with dialogs being annotated for a total of 13 action units and three additional categories not captured by FACS (head shakes and nods, and asymmetrical eyebrows). For a more detailed description of the data, see Louwerse et al. (2012).

### Evaluation approach

To minimize the influence of a Zipfian pattern being driven by other factors than the linguistic units extracted from speech, such as corpus size or frequency, we created smaller samples of utterances. Note that this procedure was slightly different for the nonverbal units, and the explanation below does not apply to them. The sample creation for the nonverbal units is outlined later in this section. The purpose of taking these samples was to create multiple sets of observations from within one corpus so that multiple *α* values of a corpus could be compared. Depending on the size of the corpus this yielded 1–205 samples (see Tables 2, 3 and 4). Utterances were chosen as they are the minimal communicative unit from which the observations of linguistic units can be extracted. All utterances within a corpus were put on a large pile. From this pile, we created samples of 2,500 utterances each, by random sampling (without replacement). A sample size of 2,500 utterances was chosen as a trade-off between the number of samples, while maintaining an individual sample size that was sufficiently large to fit Zipf’s law on. As a consequence of this choice, each corpus, with the exception of the Griffith Corpus of Spoken Australian English (which consists of one sample, given its small size), could be split up into at least two samples, benefitting the reliability of the analysis. For each utterance in each sample, we then extracted the linguistic units.

**Table 2:** Overview of the average α values for each corpus and word and utterance level unit pairs

		Samples	Word Unigrams	Word Bigrams	First Word	Last Word	Utterance	Utterance Length
Dialog Type	Corpus	*n*	*α*	*α*	*α*	*α*	*α*	*α*
Mixed	Santa Barbara Corpus	16	0.895^c^	0.559^a^	0.915^b^	0.761^a^	0.994^a^	1.153^a^
Face-to-face	Croatian Spoken Language Corpus	23	0.894^b^	0.563^a^	0.943^b^	0.722^a^	0.865^a^	0.988
	Griffith Corpus of Spoken Australian English	1	0.902^c^	0.575^a^	0.965^a^	0.775^b^	1.021^b^	1.096^a^
	Spoken Dutch Corpus							
	Face-to-Face Section	205	0.932^a^	0.567^a^	1.113^a^	0.984^b^	1.416^a^	1.107^a^
	ORAL2013 Corpus	157	0.935^a^	0.608^a^	1.009^a^	0.900^a^	1.150^a^	0.652^b^
	Garvey Corpus	4	0.862^b^	0.541^a^	0.873^b^	0.745^a^	0.861^a^	1.073^c^
Telephone	Spoken Dutch Corpus							
	Telephone Section	86	0.947^b^	0.603^a^	1.173^a^	1.042^a^	1.370^a^	1.117^a^
	Switchboard Corpus	122	0.928^c^	0.625^a^	1.088^b^	0.936^a^	1.277^a^	1.119^a^
	CallFriend							
	English	8	0.899^b^	0.600^a^	0.979^b^	0.788^a^	0.914^a^	1.068^b^
	Farsi	12	0.853^c^	0.494^a^	0.927^a^	0.844^a^	1.050^a^	0.907^b^
	Japanese	12	0.963^b^	0.688^a^	1.199^a^	1.126^a^	1.433^b^	1.134^a^
	Korean	16	0.821^a^	0.454^c^	1.106^a^	1.011^a^	1.539^a^	1.146^a^
	Spanish	33	0.940^b^	0.581^a^	0.962^b^	0.841^a^	1.107^a^	1.055^a^
	CallHome							
	Arabic	12	0.894^a^	0.506^a^	1.028^a^	0.912^a^	1.229^a^	1.089^a^
	English	13	0.911^b^	0.614^a^	0.999^b^	0.828^a^	1.067^a^	1.078^a^
	German	12	0.937^b^	0.604^a^	1.093^a^	0.971^a^	1.252^a^	1.048^a^
	Japanese	15	0.933^a^	0.615^a^	1.207^a^	1.153^a^	1.542^a^	1.232^a^
	Mandarin	15	0.886^c^	0.513^a^	1.003^c^	0.968^a^	1.109^b^	1.068^a^
	Spanish	12	0.942^b^	0.579^a^	0.978^b^	0.860^a^	1.059^b^	1.036^a^
Task-based	AMI Meeting Corpus	51	0.925^c^	0.590^a^	1.062^a^	0.953^a^	1.249^a^	1.222^a^
	ICSI Meeting Corpus	43	0.927^c^	0.631^a^	0.971^b^	0.891^a^	1.149^a^	1.061^a^
	HCRC Map Task Corpus							
	Eye Contact and Familiarity	2	0.926^a^	0.671^a^	0.975^a^	0.940^a^	1.263^a^	1.230^a^
	Eye Contact and No Familiarity	2	0.937^a^	0.694^a^	1.010^a^	0.970^a^	1.260^a^	1.243^a^
	No Eye Contact and Familiarity	3	0.936^a^	0.687^a^	1.026^a^	1.012^a^	1.301^a^	1.275^a^
	No Eye Contact and No Familiarity	2	0.940^a^	0.699^a^	1.027^a^	1.020^a^	1.271^a^	1.254^a^
	TRAINS93 Corpus	2	0.903^b^	0.699^b^	1.159^a^	0.955^a^	1.272^a^	0.999^a^
	Walking Around Corpus	7	0.945^b^	0.667^b^	1.162^a^	0.944^a^	1.326^a^	1.089^a^

^a^ = *R*^2^ > .9, ^b^ = *R*^2^ > .8, ^c^ = *R*^2^ > .7

**Table 3:** Overview of the average α values for each corpus and syntactic level unit pairs

		Samples	PoS Unigrams	PoS Bigrams	First PoS	Last PoS	PoS Sequence	Content Count	Function Count
Dialog Type	Corpus	*n*	*α*	*α*	*α*	*α*	*α*	*α*	*α*
Mixed	Santa Barbara Corpus	16	0.837^b^	0.870^b^	1.062^b^	1.100^b^	1.117^a^	1.442^a^	1.427^b^
Face-to-face	Griffith Corpus of Spoken Australian English	1	0.839^c^	0.864^b^	1.340^a^	1.169^b^	1.197^a^	1.275^a^	1.337^b^
	Spoken Dutch Corpus								
	Face-to-Face Section	205	0.721^b^	0.835^a^	1.275^a^	1.140^a^	1.239^b^	1.277^a^	1.220^c^
	Garvey Corpus	4	0.969^c^	0.923^c^	1.283^a^	1.221^a^	0.959^a^	1.381^c^	1.658^a^
Telephone	Spoken Dutch Corpus								
	Telephone Section	86	0.734^c^	0.835^a^	1.500^a^	1.288^a^	1.354^a^	1.262^a^	1.175^c^
	Switchboard Corpus	122	0.804^b^	0.871^b^	1.246^b^	1.102^b^	1.362^a^	1.355^a^	1.233^b^
	CallFriend English	8	0.864^b^	0.863^b^	1.252^a^	1.053^c^	1.118^a^	1.361^b^	1.382^b^
	CallHome English	13	0.826^b^	0.864^b^	1.267^a^	1.010^c^	1.311^a^	1.284^a^	1.215^b^
Task-based	AMI Meeting Corpus	51	0.805^c^	0.854^b^	1.413^a^	1.253^b^	1.460^a^	1.411^a^	1.200^b^
	ICSI Meeting Corpus	43	0.743^c^	0.873^b^	1.153^a^	1.125^a^	1.310^a^	1.277^a^	1.069^b^
	HCRC Map Task Corpus								
	Eye Contact and Familiarity	2	1.062^b^	0.982^a^	1.307^a^	1.526^b^	1.450^b^	1.571^a^	1.074
	Eye Contact and No Familiarity	2	1.079^b^	0.995^a^	1.350^a^	1.573^a^	1.506^a^	1.537^a^	1.019
	No Eye Contact and Familiarity	3	1.046^c^	0.972^a^	1.344^a^	1.563^a^	1.518^a^	1.587^a^	1.086
	No Eye Contact and No Familiarity	2	1.063^c^	0.981^a^	1.367^a^	1.556^a^	1.558^a^	1.568^a^	1.012
	TRAINS93 Corpus	2	0.764^c^	0.850	1.544^a^	1.324^a^	1.484^a^	1.174^a^	0.949^c^
	Walking Around Corpus	7	0.840	0.919^b^	1.713^a^	1.351^a^	1.453^b^	1.266^a^	1.134^b^

^a^ = *R*^2^ > .9, ^b^ = *R*^2^ > .8, ^c^ = *R*^2^ > .7

**Table 4:** Overview of the average α values for the dialog act unigram distribution of each corpus

	Samples	Dialog Acts	Dialog Act Unigrams
Corpus	*n*	*n*	*α*
Switchboard Corpus	122	42	1.547^a^
AMI Meeting Corpus	51	15	1.163^a^
ICSI Meeting Corpus			
General Tags	43	11	2.148^a^
Specific Tags	43	41	0.927^b^
Full Tags	43	2,052	1.219^a^
All Tags Split	43	56	1.344^a^
HCRC Map Task Corpus			
Eye Contact and Familiarity	2	13	0.705^a^
Eye Contact and No Familiarity	2	13	0.826^a^
Eye Contact and Familiarity	3	13	0.726^a^
No Eye Contact and No Familiarity	2	13	0.791^a^
Memphis Multimodal Map Task Corpus	2	13	1.255^c^

^a^ = *R*^2^ > .9, ^b^ = *R*^2^ > .8, ^c^ = *R*^2^ > .7

Since the linguistic units operated on different levels (e.g., word level to utterance level), the total number of observations differed for each unit. For most linguistic units, one observation was extracted from each utterance, leading to a total frequency of 2,500 observations in each sample (because each sample contained 2,500 utterances). This was the case for the first word of the utterance, the last word, the utterance length, first PoS, last PoS, PoS sequence, content count, function count, and dialog act unigrams. After all, for all these linguistic units, there is only one observation per utterance. These 2,500 observations were then used to create the frequency distribution. The other linguistic units (word unigrams and bigrams and PoS unigrams and bigrams) naturally could contain more than one observation per utterance (i.e., as many as the number of words or word bigrams in the utterance). Hence, the token frequency for these units differed across samples.

The somewhat arbitrary decision to select sample sizes of 2,500 utterances could be argued to bias our findings. We would argue that a considerably smaller sample size (e.g., 100 utterances) may bias the findings and that too few samples will bias the generalizability of our findings. Nevertheless, to demonstrate that the effects of different sample sizes on the resulting parameter estimates of Zipf’s law are rather minimal, we randomly sampled 10,000 utterances from the Switchboard Corpus. From these 10,000 utterances, we then sampled 2,500 utterances and subsequently from the 2,500 utterances, we sampled 1,000 utterances. For each of these three sets, we then extracted the utterance unit frequencies and fitted Zipf’s law to the resulting distribution. The utterance unit was chosen because the size of each countable linguistic unit is the largest across all linguistic units—namely, the entire utterance, resulting in the lowest number of observations in a single sample across the units—namely, as many as the sample size, and hence arguably the first unit for which we might expect to observe an influence of a sample size that is too small. The estimated *α* values were from the largest to the smallest sample size: 1.28, 1.27, and 1.25, while the *R*^2^ determination coefficients were .91, .90, and .91. In other words, a sample of 10,000, 2,500, or 1,000 would presumably yield similar results, except that the largest sample would decrease the generalizability and the smallest sample size might bias findings (with 1.25 being the lowest value).

**Fig. 2:**
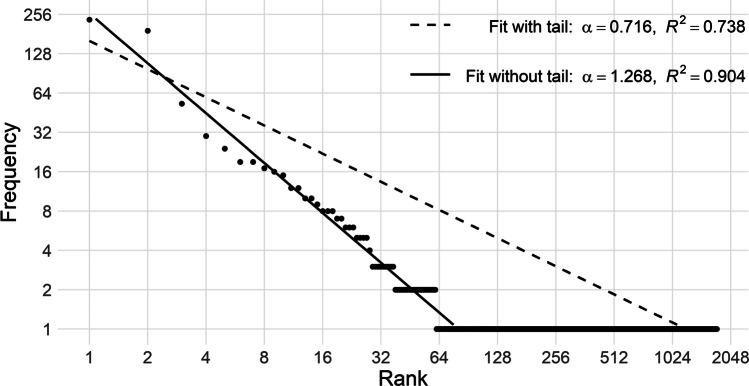
Influence of a long tail of an utterance distribution on the fit with Zipf’s law. *Note.* This graph shows two fits of Zipf’s law on the same sample of a distribution of utterance units from the Switchboard Corpus. The dashed line represents the fit where the parameters of Zipf’s formula were estimated with the inclusion of all items, while the solid line represents the fit where the parameters were estimated with the removal of all items that only occurred with a frequency of one in that sample, the latter leading to a higher *α* and *R*^2^. This behavior of the fits with Zipf’s law are representative for the behavior of utterance and PoS sequence distributions across all samples and corpora

Because resulting frequency distributions of some linguistic units had a long tail of observations only occurring (i.e., most notably for the utterance and PoS sequence distributions), we removed all observations that only occurred once to avoid a forced power law driven by the tail of the distribution rather than by the slope of the curve. As with choosing the sample size, here, too, questions about this decision could be raised, and here again we would argue that *not* making this decision would bias our findings. To illustrate the effect of removing the observations that only occurred once, we use the Switchboard Corpus. In Fig. 2, we fitted Zipf’s formula to the distribution including and excluding items with frequency one. We can immediately see that the items including the tail have a major impact on the slope of the distribution and thus on the parameters of Zipf’s law, as confirmed by the observed *α* values. Unsurprisingly, the goodness of fit is significantly worse in the distribution that includes all items. In addition, cutting off the tail is a practice that is regularly done in fitting Zipf’s law because of the larger variability in frequencies at the tail of the distribution (Clauset et al., 2009; Piantadosi, 2014).

It may be worth emphasizing that it is impossible to rule out any effect of frequency, as frequency—in relation to rank—is the very nature of Zipf’s law. However, we have tried to reduce those effects that can be attributed to frequency to a maximum extent in a number of ways. Whereas for the linguistic units with one observation per utterance (e.g., first word and PoS sequence), thus adding up to exactly 2,500 observations per sample, frequencies cannot explain any differences in *α* values, because frequencies across samples (and corpora) are equal. For the linguistic units that do not necessarily extract one observation per utterance and hence do not add up to 2,500 observations per sample (e.g., word unigrams and PoS bigrams) frequency does, however, unlikely have an effect. First, because we kept the size of each sample in terms of number of utterances the same, any differences in frequency of these units must be small. Secondly, because we cut off the tail of the distribution, small frequencies cannot drive the curve of the distribution. Differences in types and tokens, however, are unlikely to explain the alpha values either, because due to the skewedness of the Zipfian distribution, the majority of the tokens in any corpus are captured by a minority of the types. As a consequence, more frequent linguistic units are automatically more robust against small changes in their frequencies across corpora.

For the nonverbal channels in the AMI Meeting Corpus and the Memphis Multimodal Map Task Corpus, we processed the units slightly differently, because this data could not be divided into utterances. Hence, instead of first creating one large set of utterances, we created one large set of observations (e.g., hand gestures) directly from each of the nonverbal units. From this pile of observations, we then created the samples of 2,500 observations. This means that the number of observations in each sample from which the frequency distributions were created, was equally large, namely 2,500.

After having created the 2,500 observations samples and having removed the tail of the distribution by eliminating observations with a frequency of 1, we created the frequency distributions from the observations in each sample and fitted the Zipf formula using maximum likelihood estimation. Finally, we observed the *R*^2^ determination coefficient and the steepness of the curve, quantified by *α*.

### Results

We analyzed a total of 28 dialog corpora subdivided into 288 corpus-linguistic unit pairs. For each individual sample of each corpus-linguistic unit pair, we computed the *R*^2^ and *α* values. A summarization of these values through averaging the values for each corpus–unit pair is presented in Tables 2, 3, 4 and 5.

**Table 5:** Overview of the average α values for the nonverbal frequency distributions

		Tokens	Types	Samples	Nonverbal Unigrams
Corpus	Channel	*n*	*n*	*n*	*α*
Ami Meeting Corpus	Head Gestures	7,110	7	2	1.691^b^
Memphis Multimodal Map Task Corpus	Eye & Facial Gestures	33,062	19	13	1.434^c^
	Hand Gestures	6,631	8	2	2.219^c^

^a^ = *R*^2^ > .9, ^b^ = *R*^2^ > .8, ^c^ = *R*^2^ > .7

The vast majority of all units for the vast majority of all corpora yielded a goodness of fit *R*^2^ value for Zipf’s law that was high. Of the 288 average *R*^2^ values reported in Tables 2, 3, 4 and 5, we found that 71% yielded an *R*^2^ > .9, 91% yielded an *R*^2^ > .8 and 99% yielded an *R*^2^ > .7. This is in line with the high *R*^2^ values generally reported in the literature for word unigrams (Piantadosi, 2014; Ridley, 1982). Not one linguistic or nonverbal unit showed a mediocre or poor fit, demonstrating that when solely looking at the *R*^2^ values, Zipf’s law would seem to be present at all levels within dialog, including nonverbal channels and intentional dialog acts. Also, the distribution over utterance lengths, despite the distribution not being strictly ordered from “large” to “small,” showed a good fit to Zipf’s law, a result also previously reported in (Linders & Louwerse, 2020). This also holds when dividing the groups into function words and contents words.

The *R*^2^ values across corpora showed no uniform relationship to the underlying *α* values that we hypothesized to provide more insights into Zipf’s law. A Spearman correlation between *α* and *R*^2^ on all samples, per linguistic unit, did yield a significant correlation between *α* and *R*^2^ for the majority of the linguistic units, but this correlation was generally moderate (*M* = .23), with some notable exceptions (*r* = −.46 for word bigrams*, r* = −.36 for last word, *r* = −.61 for PoS bigrams, *r* = .70 for utterance length, *r* = .72 for dialog acts). For completeness, these correlations are shown in Table A1 of the Supplementary Material.

Contrary to the findings in the *R*^2^ values, when looking at the *α* values in Tables 2, 3, 4 and 5, we can observe considerably larger fluctuations between different linguistic units, as well as between different corpora within a linguistic unit. Most notably, *α* is low for word bigram distributions with values around 0.6 and for PoS unigrams distributions with values around 0.8. Larger fluctuations in *α* between corpora can be observed for the distributions of utterances and dialog acts, while for word unigrams, this fluctuation seems considerably smaller. We do not have an explanation why for some linguistic units the fluctuations between corpora are larger than for others. Vocabulary size (number of types or ranks) is not the reason, as this has no consistent effect on *α* for the word level and utterance level units.^4^ For completeness, the table with correlations is shown in Table A2 of the Supplementary Material.

Overall, we can conclude that across the different linguistic units across the different dialog corpora, and across the different languages good fits to Zipf’s law can be observed with minimal differences in *R*^2^, while more variance in *α* can be observed. This is insightful in itself, as very few studies have looked at dialog corpora, and no study has presented a comprehensive overview. However, these overall goodness of fit results say little about the mechanisms behind Zipf’s law, something we will address in the second part of this study.

## The cognitive effort hypothesis

### Theoretical framework

It is worth recalling the cognitive effort hypothesis that we introduced. If the *α* value is larger, it suggests speakers are more economical in their linguistic contributions. If *α* is smaller, it suggests speakers are less economical. The economy of the linguistic contributions may be driven by cognitive resources being available. That is, the higher the extralinguistic cognitive load, for instance, due to the cognitive resources required for a particular cognitive task, the more economical a speaker needs to be, yielding larger greater *α* values.

Most research has focused on evidence for the presence of Zipf’s law in language rather than trying to explain the cause of this regularity. Zipf (1949) proposed the principle of least effort as an explanation, but this hypothesis has hardly been formalized or empirically validated. Ferrer-i-Cancho’s (2005, 2006, 2018) information theoretic model is a notable exception. This model showed that, under the plausible assumption that words have meanings in the form of associations, Zipf’s formula can be instantiated with a communication optimization function from information theory. This communication optimization function consists of two parts. On the one hand, there is a pressure to maximize the expressivity of the system, while on the other hand a pressure of parsimony tries to limit the cost of communication. Instead of an economization of the linguistic effort by both the speaker and hearer separately, as Zipf (1949) proposed, this approach, also consisting of two opposing pressures, is formalized in terms of maximizing information transfer while keeping the cost low. A parameter is used for balancing these two pressures. Ferrer-i-Cancho (2018) argued that both the speaker and hearer benefit from each of the two pressures in the model, hence not making any distinction between the speaker and hearer in the hypothesized communicative pressures.

While the model is not yet a fully-fledged theory, it is able to pose hypotheses and make predictions. These predictions include the range of plausible exponents for word frequency distributions being limited to a small interval of *α* ≈ 0.7 and *α* ≈ 2 (Ferrer-i-Cancho, 2005) and communication breaking down when the exponent becomes too small (Ferrer-i-Cancho, 2006). Moreover, the model is able to predict how different balances of the two pressures affect the exponent of Zipf’s law. The parameter that balances these pressures, represents the degree of optimization of the language system. If language is more balanced toward maximizing information transfer, it will suffer more on the cost, associated with the communication, which translates itself into a lower value for the exponent *α* (Ferrer-i-Cancho, 2005). Vice versa, if the system is more balanced toward minimizing the cost of the conversation, it will compensate this by losing some expressivity, which translates itself into a higher value for *α*. These predictions seem very consistent with the cognitive effort hypothesis we propose here. In fact, the parameter balancing the opposing pressures can to some extent be interpreted as the degree of cognitive effort (Ferrer-i-Cancho, 2018). However, what distinguishes the predictions that this theory makes from the predictions that our proposed cognitive effort hypothesis makes, is that, like the predictions from Zipf’s principle of least effort, cost or effort is not necessarily restricted to cognitive effort in Ferrer-i-Cancho’s model. In this study we do however limit ourselves to investigating cognitive effort.

The cognitive effort hypothesis is not precise enough yet to yield predictions in terms of an exact mathematical formula, but it is capable of yielding predictions for the simplest scenario, a binary decision on the effect on *α*, given a certain condition. In Table 1 we identified the corpora on five dimensions: face-to-face, familiarity, task-based, adults, and strict roles. The purpose of using these dimensions was to demonstrate the heterogeneity of the dialog corpora that Zipf’s law applies to. These dimensions, however, also offer insight in the cognitive effort hypothesis, as it allows a first exploratory analysis on the effect of these dimensions on the *α* values by dummy-coding these dimensions (Cohen et al., 2003) and applying a statistical analysis. But before we can proceed with the statistical analysis, we have to specify in more detail the predictions the cognitive effort hypothesis yields on *α* for each of the dimensions.

In face-to-face conversations, one can make use of behavior channels on multiple modalities, including visual modalities, such as eye contact and gestures, to communicate information (Clark, 1996). In scenarios such as telephone conversations, one can simply not use these visual behavior channels and thus must rely only on using speech. This lack of visual information imposes constraints on the conversation and in turn on the cognitive load: it is easier to communicate when one can use the visual modalities. Indeed, a study on the HCRC Map Task Corpus revealed that the lack of eye contact influenced efficient information transfer, leading to turns with more words and more interruptions (Boyle et al., 1994). Instruction followers also compensated the lack of visual feedback through increased use of back channels. Hence, the dialog participants had to put more effort into their linguistic contributions as a consequence of a lack of visual information. Another study, on the same corpus, showed that in the dialogs where participants could not make eye contact, there was an increase in disfluencies (Branigan et al., 1999), which might indicate an increase in planning effort for the linguistic contribution, and thus cognitive effort (Lickley, 2001). In line with a cognitive effort hypothesis, we therefore predicted lower *α* values in conversations that took place in situated environments where dialog participants could see each other, than in conversations where no visual channels could be used for communication. In short, we expected *α* to correlate negatively with the presence of a face-to-face setting.

Just like face-to-face communication allows for offloading cognitive resources that cannot be offloaded in telephone conversations, we predicted that if participants knew each other they could also offload cognitive resources. After all, when participants know each other, they can rely on common ground and they already have an impression how the other might respond in the conversation. In addition, Bard et al. (2002) found higher disfluency rates in dialogs of the HCRC Map Task Corpus where participants did not know each other, which again could be taken as a proxy for the degree of planning of linguistic contributions (cf. Lickley, 2001). Dialog participants that were familiar with each other, are then expected to have lower cognitive load imposed on them, yielding higher *α* values. Thus, a negative correlation between the presence of familiarity in the dialogs and the *α* value was expected.

In task-based corpora, the communication is focused on the task. Therefore, the largest cognitive load is arguably spent on the task, compromising cognitive effort spent on the communication. Hence, we expected speakers to become more economical in task-based conversations with higher expected *α* values in task-based conversations than in non-task-based settings. So, we expected a positive correlation between the presence of a task in the dialogs and *α*.

In a similar vein, for those corpora in which strict roles were identified for the dialog participants (i.e., instruction giver and instruction follower), we predicted speakers to be more restricted in their linguistic freedom due to their strict role. This higher cognitive effort would then be compensated with a more economic linguistic contribution, thus yielding a higher expected *α* value than in conversations where speakers were less restricted in a particular role. Again, we expected a positive correlation between the presence of dialogs where the participants have strict roles and *α*.

One corpus in our collection did not include adults, but children. During their development children are developing their language skills. Next to not fully developed vocabulary sets, which could have an effect on the resulting frequency distributions (Segbers & Schroeder, 2017), children arguably require more cognitive effort in conversations. Indeed, Baixeries et al. (2013) found that as children grew older and progressed their language skills, the *α* values of the resulting frequency distributions became smaller. The authors attributed the decrease in *α* to an increase in the linguistic complexity of the communicative system. This hypothesis is intimately related to the cognitive effort hypothesis proposed in the current study, as the linguistic complexity arguably is a measure of the available cognitive resources in the conversation. More cognitive resources available for the conversation, allows for using more complex linguistic structures. Because we only included one corpus with children’s speech and because all corpora differ in more than one dimension from each other, we could not reliably compare the effect of participants with fully developed language skills versus participants with not fully developed language skills on *α*.

### Testing the cognitive effort hypothesis

#### Methods

In order to test the cognitive effort hypothesis, we would need to confirm that there is a significant difference between a hypothesized higher cognitive effort condition and a hypothesized lower cognitive effort condition, and also that the former condition has a higher average *α* value. For each linguistic unit we performed a Mann–Whitney *U* test, with the *α* values as dependent variable and the binary dimensions, specifying each corpus (face-to-face, familiarity, task-based, and strict roles) as independent variables. Because the *α* values were not normally distributed, we resorted to a nonparametric test.

There is only one corpus with conversations involving children. This means that we only have one configuration of dummy-encoded dimensions that includes the absence of the adults dimension, making it virtually impossible to reliably measure the influence of the presence of the adults dimension. For this reason, we removed this corpus. Similarly, because the Santa Barbara Corpus does not contain a homogenous set of dialogs, it was also removed from the analysis. We also only performed the analysis for the English corpora because of potentially relevant differences in *α* values across languages, and because the other languages only cover half of the dimensions with at most two corpora for each language. Finally, we did not include the pragmatic and nonverbal units in our analysis for the same reason of data sparsity.

#### Results

The differences in *α* values between the conditions (e.g., familiar vs. unfamiliar) of each dimension (face-to-face, familiarity, task-based, strict roles) with their associated significance levels, calculated using the Mann–Whitney *U* test, are shown in Table 6. It is important to state that this analysis is illustrative given the number of cases and the differences across corpora. Nonetheless, Table 6 gives insight in the influence of a dimension on the *α* value particularly in relation to the cognitive effort hypothesis. Recall that the cognitive effort hypothesis predicts higher *α* values with higher cognitive effort for instance due to the task at hand, and lower *α* values when more cognitive resources are available for instance due to the fact that participants know each other or can see each other. Hence, a higher *α* value suggests speakers are more economical in their linguistic contribution and use less cognitive effort on the linguistic unit, for instance because the cognitive effort is taken up by other reasons such as the effort on the task at hand.

**Table 6:** Comparison of mean α values for each linguistic unit and each corpus-specific dimension

		Face-to-face		Familiar		Task-based		Strict roles	
		True	False	True	False	True	False	True	False
Level	Linguistic Unit	*M*	*M*	*M*	*M*	*M*	*M*	*M*	*M*
Word	Word Unigrams	0.923	0.925	0.921	0.929^**^	0.927	0.921	0.935^**^	0.923
	Word Bigrams	0.609	0.628^**^	0.609	0.632^**^	0.620	0.618	0.681^**^	0.614
	First Word	1.013	1.074^**^	1.010	1.092^**^	1.031	1.061^**^	1.086^*^	1.043
	Last Word	0.917	0.918	0.901	0.939^**^	0.932^**^	0.904	0.967^**^	0.914
Utterance	Utterance	1.190	1.234^**^	1.161	1.280^**^	1.218^**^	1.210	1.295^**^	1.207
	Turn Length	1.148	1.114	1.137	1.120	1.150^*^	1.109	1.161	1.127
Syntactic	PoS Unigrams	0.796	0.822^**^	0.805	0.817^**^	0.803	0.817^**^	0.942^**^	0.799
	PoS Bigrams	0.870	0.877^**^	0.869	0.879^**^	0.876^*^	0.872	0.942^**^	0.868
	First PoS	1.295	1.285	1.290	1.289	1.329^**^	1.251	1.509^**^	1.270
	Last PoS	1.209	1.126^**^	1.181^**^	1.143	1.236^**^	1.093	1.450^**^	1.139
	PoS Sequence	1.376	1.355	1.354	1.378	1.406^**^	1.323	1.485^**^	1.354
	Content Count	1.358	1.349	1.353	1.353	1.359	1.348	1.407	1.349
	Function Count	1.159	1.223^**^	1.179	1.212^**^	1.129	1.258^**^	1.072	1.204^**^

The significance levels are indicated by ^*^p < .05, ^**^p < .01. A single Mann–Whitney *U* test was performed for each linguistic unit to investigate whether the *α* values of both conditions differed significantly. For better readability, the significance levels are indicated in the cell of the condition with the higher mean value

When considering the most commonly used linguistic unit, word unigrams, the dimensions familiarity, and strict roles yield the clearest effects, while the other dimensions do not show a significant difference between the conditions. When the dialog concerns participants that are familiar with one another, more cognitive effort can be placed on the linguistic units, leading to lower *α* values. However, when the dialogs concern strict roles, the effort shifts to the task of the communication, reducing cognitive effort on the linguistic units as exemplified by the increased *α* values. These patterns are more or less consistent across the other linguistic units. The least consistent is the task-based dimension with significantly larger *α* values for some linguistic units and also significantly smaller *α* values for other units. Because the corpora with strict roles is a subset of the task-based corpora, it is interesting to observe that the task-based dimension does not show uniform patterns, while the strict roles dimension does. This might indicate that meeting dialog does not impose significant cognitive effort on the participants, compared with spontaneous free-flow dialog, but more goal-oriented dialog where participants have clearly defined roles does. The face-to-face and familiarity dimensions show very consistent results, with all significant effects being negatively correlated with *α*. The same is true for the strict roles dimension, with the exception of the function count unit. A possible explanation is that function words might be used more extensively and with more variability in situations that require more cognitive effort, because they are easier to retrieve, might give more structure to the utterance, might give the speaker more time to formulate the remaining part of their utterance and some classes of function words, such as adpositions and conjunctions can be omitted to be more efficient (Yung et al., 2017).

The results show general consistency with the cognitive effort hypothesis in this overview. However, the overview analysis should be considered rather illustrative because it can be argued to be problematic. The set of English corpora used in this analysis is small and differ on multiple dimensions, some that are difficult to compare. In addition, the variation in *α* is large. There are also differences in annotation and different dialects of English that are used. Hence, it is very likely we are committing a Type I error, finding effects that are not there. For this reason, we performed an additional analysis in a more controlled setting, where we tried to minimize for a Type I error. This analysis, which we will refer to as the minimal pair analysis, will be described next.

### Minimal pair analysis

As stated before, the overview analysis is illustrative, but shows patterns that are worth pursuing in light of the cognitive effort hypothesis. Because of the problems with the overview analysis, we also performed a more reliable but smaller-scale analysis where we compared samples of speech that differed minimally. In the corpora that we acquired in the first part of the paper, we identified corpora or turn segments within corpora that differed minimally from each other, with the only difference being a clearly hypothesized difference in cognitive effort. So, the minimal pair analysis could shed more light on the cognitive effort hypothesis with a higher precision as the conditions are highly controlled.

#### Corpus pairs

For a minimal pair analysis, we split the number of corpora, according to our hypotheses, into higher and lower cognitive effort conditions. In the higher cognitive effort condition, we expected speakers to economize more on their linguistic contribution, leading to higher *α* values, while in the lower cognitive effort condition, we expected speakers to economize less on their linguistic contribution, leading to lower *α* values. We will start off with describing the minimal pairs that we extracted from the collected set of corpora. For consistency, the condition with the hypothesized higher economization is written first and always followed by the condition with the hypothesized lower economization.

##### Instruction giver – instruction follower.

The HCRC Map Task Corpus and the Walking Around Corpus have a similar setup, with an instruction giver (IG), giving details about the route to be followed, while an instruction follower (IF) had to use the instructions to find or draw the route. Because both participants in the task had very different roles, they can be contrasted in a controlled setting. We split the speech from the instruction giver and the instruction follower and analyzed both parts separately on the *α* values associated with the fit of the Zipf formula. An analysis of disfluency rates on the HCRC Map Task Corpus showed that instruction givers have higher disfluency rates than instruction followers, which was attributed to more involved planning of the route and of the linguistic contribution (Branigan et al., 1999). Hence, the instruction giver might have a higher overall cognitive load, but also might have to allocate significant cognitive resources to the planning of the linguistic contribution, which is far less important for the instruction follower. It is far more important for the instruction follower to allocate cognitive resources to the task than to their linguistic contributions. Hence, we predicted that the instruction follower would allocate fewer cognitive resources to their linguistic contribution than the instruction giver, yielding higher *α* values for the instruction follower than the instruction giver. In the case of the Walking Around Corpus, the instruction follower needed to physically walk the route, which arguably takes up even more cognitive resources than marking the route on a map.

##### Backward–forward communicative functions.

The Switchboard Corpus was annotated with dialog acts. These dialog acts can be divided into forward (Fwd) communicative functions and backward (Bwd) communicative functions (Jurafsky et al., 1997).^5^ Forward communicative functions add new information to the dialog, while backward communicative functions respond to something that has been said before in the dialog. A similar hypothesis can be posed as for the previous corpus pairs. Since backward functions do not pose any new information and respond to something that has been said previously, there is a focus on processing information and less on communicating, while, in general, forward communicative functions focus more on the language and communication and less on comprehension. Hence, we expected more economization for utterances with a backward communicative function than for utterances with a forward communicative function, resulting in higher *α* values for the set of utterances with a backward communicative function, as compared with the set of utterances with a forward communicative function.

##### No eye contact–eye contact in conversations.

The HCRC Map Task Corpus was designed with two additional experimental conditions in mind—eye contact and familiarity—and was equally balanced for each of the four resulting variable pairs. The participants were also balanced for each condition, since each participant took part in two dialogs, each with different conditions on eye contact and familiarity. This controlled setting allowed us to investigate the effects of eye contact on the *α* values. As argued before, we expected that the lack of communication through the visual channels increases the cognitive effort needed in the conversation, leading to economization in the speakers’ linguistic contributions.

##### No familiarity–familiarity in conversations.

Similarly for familiarity, there was high experimental control over both conditions in the HCRC Map Task Corpus, allowing us to also contrast these conditions. Again, as argued before, we expected dialog participants that were not familiar with each other to have put more cognitive resources into knowing and understanding each other, resulting in more economization on the linguistic contributions of speakers.

##### Telephone–face-to-face conversations.

Finally, the Spoken Dutch Corpus contains both telephone and situated face-to-face conversations between family members and friends. These sections differ only in one condition, which is whether the dialogs were situated and thus whether the participants could use visual channels to communicate information. As previously argued, because of the lack of visual information in telephone conversations, we hypothesized that speakers would economize their linguistic contribution more, in turn leading to higher *α* values for the telephone conversations.

#### Evaluation approach

We used the sampled data that we have used throughout this article for this analysis as well. For the analysis of the instruction giver and follower and the forward and backward communicative functions, we divided the observations of each sample into one of the two conditions because both conditions could be observed within the same sample and the division between conditions is on an utterance level. We noticed that the number of observations for many linguistic units differed considerably and consistently for the two conditions where we split within each sample, which, in turn, might affect the *α* values. Hence, we equalized the number of observations for each sample and unit for both conditions, by randomly sampling once more from the sample of the condition that has a larger number of observation. We sampled as many utterances from the condition with the most observations as were in the condition with the least observations. For the analysis of backward and forward communicative functions in the Switchboard Corpus, the sample sizes became very small, because a third group of dialog acts that could not be classified into a forward or backward communicative function was discarded. Therefore, we decided to combine two samples, reducing the total number of samples from 86 to 43, but increasing the size of each sample. A very similar approach was taken for the minimal pairs where the conditions were extracted from different corpus sections. In those cases, we could not divide the observations within a sample into one of the conditions. As a consequence, there might be slight differences in the average number of observations per sample between the conditions. This however only applies to the linguistic units that were not sampled on an utterance level (word unigrams and bigrams and PoS unigrams and bigrams), since for the other linguistic units, one observation is sampled per utterance, leading to a fixed total of 2,500 observations in each sample for these units. Furthermore, as explained before, in the construction of the samples we have tried to control for any frequency effects as much as possible by taking the same number of utterances across all samples and by cutting off the tail. As a consequence, any differences in the total number of observations between samples are small. Hence, we think it is unlikely that frequency effects could drive any of the findings.

To analyze whether there were systematic and significant differences between the *α* values of the samples from the higher cognitive effort condition and the *α* values of the samples from the lower cognitive condition, we performed an independent samples *t*-test. Because the *α* values are approximately normally distributed, unlike the dependent variables in the overview analysis, a *t* test was the preferred method of analysis.

#### Results

The results from the minimal pair analysis are shown in Table 7. The average *α* values are reported for both conditions of each pair, as well as whether they are significantly different from another. For consistency in reporting the results and for better readability, the condition presented on the left of the two condition columns is always the condition with the hypothesized lower *α* values. At the same time, we have reported the significance levels, as calculated through the *t* tests, on the condition with the higher average *α* value. As a consequence, the right condition is always hypothesized to have higher *α* values, which would indicate consistency with the cognitive effort hypothesis. For the majority of the minimal pairs and linguistic units, the condition with the higher *α* values is indeed the one we hypothesized to contain higher values, showing general consistency with our hypothesis.

**Table 7:** Overview of the *t* values for each linguistic unit and each corpus-specific dimension

	Map Task Speaker Roles		Walking Around Speaker Roles		Switchboard Communicative Functions		Map Task Eye Contact		Map Task Familiarity		Spoken Dutch Corpus Face-to-Face	
	IG	IF	IG	IF	Fwd	Bwd	True	False	True	False	True	False
Linguistic Unit	*M*	*M*	*M*	*M*	*M*	*M*	*M*	*M*	*M*	*M*	*M*	*M*
Word Unigrams	0.913	0.932^**^	0.922	0.945^**^	0.854	1.169^**^	0.932	0.938	0.932	0.939	0.932	0.947^**^
Word Bigrams	0.647	0.653	0.620	0.661^**^	0.410	0.615^**^	0.682	0.692	0.681	0.696^*^	0.567	0.603^**^
First Word	0.971	1.083^**^	1.145	1.181^**^	1.171	1.430^**^	0.993	1.027	1.006	1.018	1.113	1.173^**^
Last Word	0.902	1.080^**^	0.827	1.042^**^	0.742	1.449^**^	0.955	1.015^**^	0.983	0.995	0.984	1.042^**^
Utterance	1.233	1.282^*^	1.159	1.353^**^	0.731	1.370^**^	1.261	1.289	1.286	1.266	1.416^**^	1.370
Utterance Length	1.081	1.433^**^	0.823	1.256^**^	0.682	2.487^**^	1.236	1.267	1.257	1.248	1.107	1.117^**^
PoS Unigrams	1.041	1.106^**^	0.831	0.843	0.830	1.399^**^	1.070	1.053	1.052	1.071	0.721	0.734^**^
PoS Bigrams	0.950	0.962	0.898	0.926^**^	0.805	0.909^**^	0.988	0.975	0.976	0.988	0.835	0.835
First PoS	1.259	1.482^**^	1.729	1.718	1.299	2.142^**^	1.329	1.353	1.329	1.359	1.275	1.500^**^
Last PoS	1.456	1.698^**^	1.380	1.415^*^	1.315	1.847^**^	1.549	1.561	1.549	1.565	1.140	1.288^**^
PoS Sequence	1.410	1.652^**^	1.357	1.512^**^	0.665	1.795^**^	1.478	1.534	1.491	1.532	1.239	1.354^**^
Content Count	1.369	1.798^**^	0.973	1.481^**^	0.943	2.846^**^	1.554	1.580	1.581	1.552	1.277^**^	1.262
Function Count	0.941	1.128^**^	0.928	1.319^**^	1.178	1.909^**^	1.046	1.056	1.081^**^	1.016	1.220^**^	1.175
Dialog Acts Unigrams	0.898	1.238^**^			2.266^**^	1.811	0.766	0.752	0.717	0.809^**^		

The significance levels are indicated by *p < .05, **p < .01. A single *t* test was performed for each linguistic unit to investigate whether the *α* values of both conditions differ significantly. For better readability, the significance levels are indicated in the cell of the condition with the higher mean value

**Fig. 3:**
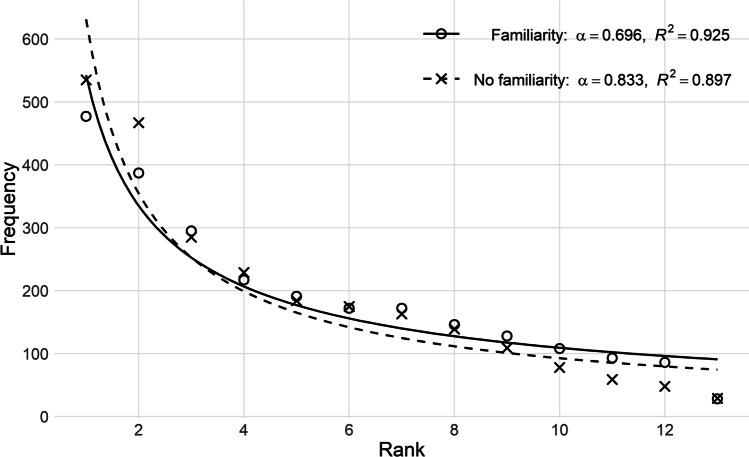
Illustration of two dialog act unigram distributions differing in the familiarity condition. *Note.* This graph shows two dialog act unigram distributions and the resulting fits of Zipf’s formula on two samples from the Map Task Corpus that differed in the familiarity condition, matching Fig. 1

In order to interpret these findings in relation to the schematic in Fig. 1, we here illustrate what a difference in *α* values signifies using the actual data. Presenting all data in a graph would require at least as many graphs as there are cells in the table, hence, we restrict ourselves to an illustration of the *α* value. For this purpose, we took the dialog act unigram distributions of two samples from the Map Task Corpus that differed in the familiarity condition. The frequency distributions and their fit with Zipf’s formula, as parameterized by *α*, of the two selected conditions are presented in Fig. 3. Figure 3 is analogous to the schematic in Fig. 1. Higher cognitive effort (i.e., when participants are not familiar with each other) yields a higher *α* value.

Zooming in on individual minimal pairs, there is a clear difference between the linguistic frequency distributions of the instruction giver and instruction follower, with the distributions for instruction follower indeed having significantly higher *α* values. This effect is similar for both the HCRC Map Task Corpus and the Walking Around Corpus, despite differences in setup, annotation (including different sets of PoS tags) and the task itself, showing the robustness of these findings.

Also comparing the backward and forward communicative functions, very significant differences can be observed. One notable exception is the dialog act unigrams, showing the opposite pattern. However, this should come to no surprise, since it is exactly on the level of dialog acts, where the utterances have been split, making this analysis virtually meaningless in this context.

The differences on the HCRC Map Task Corpus for the eye contact and familiarity conditions are smaller and not significant, indicating that the role of the speaker has a much larger influence on the frequency distributions than the setting of the dialog. The lack of large effects of these conditions could also have been caused by the task-based setting and the clear distinction in speaker roles. The task and strict roles of the participants might require significantly more cognitive resources, such that the effects of eye contact and familiarity are minimized. An explanation for the lack of significance may, however, also lie in the sample size being too small. Even though the differences are not significant in these two analyses, they are generally positive for the different linguistic units, which is still consistent with the cognitive effort hypothesis. Again, the only significant negative effect is the function count unit.

Finally, also the comparison of the telephone and face-to-face sections of the Spoken Dutch Corpus clearly show that in telephone conversations the *α* values are significantly higher for most linguistic units. The exceptions are the utterance unit and the content and function count units. An explanation for the former might be that because face-to-face conversations allow for better turn-taking coordination because of the availability of the visual channels, these conversations might be more dynamic, leading to more switching of turns (Ten Bosch et al., 2004). This dynamicity could be reflected in the utterance unit through shorter turns and thus less variability, leading to higher *α* values for this unit. We do have to note that results from this final minimal pair should be taken with more caution, because the number of conversations and topics being discussed was not controlled for and there was arguably more linguistic freedom than for the other minimal pairs. This might consequently have influenced the results, even though both sections of the Spoken Dutch Corpus have been acquired and annotated similarly. Still, the tendencies are generally consistent with the cognitive effort hypothesis.

## General discussion

A regularity in language commonly reported in computational linguistics and psycholinguistics is Zipf’s law, the inverse relationship between the frequency of a word and its rank order. The current study revisited Zipf’s law and considered it in a wider context than has generally been done in the literature by providing a critical review of the role of the law’s parameters, different linguistic units, spoken dialog and the principle of least effort in relation to the most well-known quantitative law in linguistics. We extended Zipf’s law beyond written monolog and extensively studied its application in the most natural, most direct, and most dynamic form of language use, that of spoken dialog. We did not only consider word unigram frequencies but showed that Zipf’s law has good fits to other linguistic units in spoken dialog, such as larger units of words and specific positions of words in an utterance and to units at higher linguistic and communicative levels, such as part-of-speech tags, dialog acts and nonverbal communication channels. Studying Zipf’s law in a wider context and in a form of language use that more directly operates on our cognition, also allowed us to investigate the psychological mechanisms behind Zipf’s law. Taking the principle of least effort, proposed by Zipf (1949) as a starting point, we proposed the cognitive effort hypothesis, which links the economization of the linguistic contributions of speakers to the cognitive effort necessary for extracommunicative aspects using Zipf’s law and showed evidence in favor of this hypothesis.

While the current study may seem exploratory, it is an important step toward building a more comprehensive theory of Zipf’s law and, more specifically, a psychologically plausible theory. There is a need to build theories of why and when Zipf’s law emerges in order to understand the potential and usefulness of the law (Piantadosi, 2014). Most studies on Zipf’s law have merely investigated the presence of the law in a certain distribution. While this can be very insightful, it is only the very first step in building a theory that explains the mechanisms behind Zipf’s law (Semple et al., 2022). In order to build such a theory, we need to move beyond merely establishing the law and test hypotheses that predict when the law applies and when it does not, and also test hypotheses that predict alterations in the parameters of the formula, the latter of which was attempted in this study.

Zipf’s law has generally been studied in written monolog. This can be very insightful, especially when Zipf’s law is studied from an evolutionary perspective, investigating, for example, how Zipf’s law has emerged through optimization of language over time. But in order to study the psychological mechanisms of language use and its effects on Zipf’s law, we must study Zipf’s law in a setting that more directly operates on our cognition—that of spoken language.

Furthermore, Zipf’s law has primarily been applied to word frequencies. Even though studies of Zipf’s law on word frequencies are insightful too, they are also limited in that they only focus on a very small aspect of communication. A theory of why Zipf’s law emerges that is based on cognitive factors, applied to communicative principles, such as the principle of least effort, needs to be applicable not only to word frequencies, but to other units of communication, such as syntactic, pragmatic and even nonverbal units, too. Moreover, Ferrer-i-Cancho (2018) and Semple et al. (2022) argued that any theory explaining Zipf’s law should consider a broader view. The explanation provided in the current study has shown to be robust for different registers in spoken dialog, for different linguistic units, and is general enough to eventually be extended beyond language and applied to other cognitive abilities.

Zipf’s law is often regarded as a static property—a characteristic of language. But the law seems to be at least partially dynamic, in that it can change, depending on the situation and task at hand—something that cannot be shown as easily in written language, because it is more static. The optimization benefits can, as Zipf argued, be linked to economization of a speaker’s and hearer’s efforts in conversation. This has indeed been shown to be possible in a mathematical model, making use of information theoretic notions (Ferrer-i-Cancho, 2005, 2006, 2018), but empirical evidence is scarce.

Our proposed cognitive effort hypothesis obviously ties in well with Zipf’s principle of least effort. However, even though the forces of unification and diversification, that Zipf identified as causes for observing Zipf’s law, are clearly defined in terms of cognitive effort, this is not necessarily true for all parts of the principle. For example, Zipf’s law of abbreviation relates at least partially to physical effort (Zipf, 1949). This optimization of physical effort might be due to different processes—for example, language evolution—and seems to be more static. But as we have seen, the linguistic unit that is arguably the closest to Zipf’s law of abbreviation—utterance length—showed patterns that were in line with our cognitive effort hypothesis. This is an important finding, as this means that at least part of the economic optimization that has resulted in Zipf’s law is dynamic and implies that speakers and hearers do not *actively* choose to reduce their effort, but rather that the limited available cognitive resources lead to reducing cognitive effort. And hence the economization of the linguistic contribution is at least partially due to automatic cognitive processes that are not under cognitive control of the language user.

The usefulness and explanatory power of Zipf’s law has been frequently debated because of its ubiquity in language and its mathematically simple formulation. Zipf’s law seems to apply to virtually any corpus, including randomly generated text (Li, 1992; Miller, 1957). However, what constitutes a good fit to Zipf’s law is debated and no agreement has been reached on its quantification. This study intentionally steered away from these debates. We have taken a quite liberal stance by considering only the simplest formulation of Zipf’s law as a starting point, even though it has been argued that word frequency distributions are actually more complex (Piantadosi, 2014). Even though more complex formulations of Zipf’s law are nevertheless derived from the simpler formulation, it remains to be seen how our findings extend to other, more complex formulations. In any case, different formulations of Zipf’s law would still characterize the same underlying data. Our findings are therefore very unlikely to be affected by these variations. Instead, they may have an effect of the goodness of fit, which is of no relevance to the current findings. Moreover, the consistent findings in the heterogeneous set of dialog corpora with patterns supporting the cognitive effort hypothesis cannot be dismissed as ubiquitous or merely statistical artifact of language.

The current study thus contributes to the literature that has shown support for Zipf’s law by not focusing on the statistical fit, but by investigating the mechanisms of Zipf’s law using the most natural forms of communication. By demonstrating that the steepness of the Zipfian curve may tell us more than the statistical fit and by confirming the cognitive effort hypothesis, we have not only revisited Zipf’s law, but also demonstrated its use for different linguistic units and parameters across different spoken dialogs, thereby providing a contribution for language, cognition and computation.

## Supplementary information

ESM 1 (PDF 143 kb)

## Data Availability

All data allowed to be distributed is available upon request.

## Footnotes

1 In practice, however, it can be easily estimated directly from the data. In a perfect fit, *C* is equivalent to the frequency of the observation at rank 1. In a nonperfect fit, *C* can be estimated through a least-squares regression optimization, after estimating *α*.

2 We intentionally steered away from the debate on what constitutes a separate language and kept the corpora as they were acquired, thus keeping Flemish-Dutch from Belgium and Dutch from The Netherlands in the same corpus.

3 The remaining four tags are related to the interruptions and turn abandonments and are not included in the general or specific tags as described in (Shriberg et al., 2004).

4 Because there are too few different PoS tagsets in our set of corpora, it is impossible to reliably measure the influence of the vocabulary size of the syntactic units on the *α* value.

5 We followed the division into forward and backward communicative as described in (Jurafsky et al., 1997). This division results into a third category of “other” dialog acts. In this analysis, we discard this the utterances that contain a dialog act from this group.
