# Supplementary material for: Zipf’s law revisited: Spoken dialog, linguistic units, parameters, and the principle of least effort
Source: Psychon Bull Rev. 2022 Jul 15;30(1):77–101. doi: 10.3758/s13423-022-02142-9 (PMC9971120; doi:10.3758/s13423-022-02142-9)
Supplement: Supplementary file 1 — (PDF 143 kb) [file 13423_2022_2142_MOESM1_ESM.pdf]

Supplementary Material

Table A1

Spearman Correlation between α and R^2 for each Linguistic Unit

Linguistic Unit	*ρ*
Word Unigrams	.38**
Word Bigrams	−.46**
First Word	.65**
Last Word	−.36**
Utterance	−.09**
Utterance Length	.70**
PoS Unigrams	−.25**
PoS Bigrams	−.61**
First PoS	.42**
Last PoS	.63**
PoS Sequence	.62**
Content Count	.49**
Function Count	.35**
Dialog Acts	.72**

** p < .01

Table A2

Spearman Correlation between α and the Vocabulary Size for each Linguistic Unit

Linguistic Unit	ρ
Word Unigrams	.09*
Word Bigrams	−.44**
First Word	−.45**
Last Word	−.49**
Utterance	−.54**
Utterance Length	.70**
Dialog Acts	−.05

Note. All samples for each linguistic unit were used in the correlation analysis and from each sample the number of words or dialog acts were extracted.

Because there are too few different PoS tagsets in our set of corpora, it is impossible to reliably measure the influence of the vocabulary size of the syntactic variables on the α value.

* $p < 0.05$, ** $p < 0.01$
